# Optimizing microbial cellulase fermentation with FERM-AI: risk-aware machine learning framework for fermentation and contamination prediction

**DOI:** 10.3389/fbioe.2026.1824449

**Published:** 2026-09-28

**Authors:** Ritu Chauhan, Aarushi Mishra, Megat F. Zuhairi, Vivek Kumar Gaur

**Affiliations:** 1 Artificial Intelligence and IoT Lab, Center for Computational Biology and Bioinformatics, Amity University, Noida, Uttar Pradesh, India; 2 Malaysian Institute of Information Technology, Universiti Kuala Lumpur, Kaula Lampur, Malaysia; 3 Amity Institute of Biotechnology, Amity University, Noida, Uttar Pradesh, India

**Keywords:** artificial intelligence, bioprocess optimization, cellulase fermentation, contamination risk modeling, ensemble learning, gradient boosting, machine learning, random forest (RF)

## Abstract

**Introduction:**

Stability, yield, and interactions between variables in the process, combined with the risk of contamination, pose a challenge for optimization of microbial fermentation processes. In this work, FERM-AI, a novel risk-aware machine learning surrogate framework for benchmark evaluation of cellulase fermentation, is presented, comprising of yield prediction, contamination risk assessment and an interactive decision-support interface.

**Methods:**

The framework was tested with a benchmark set of 1,000 runs of fermentation, consisting of 20 fermentation runs from experiments and 980 synthetic fermentation runs to develop the methods. Biologically relevant process optimality, stress responses and interaction effects were captured using domain-informed feature engineering, and features were then selected and expanded using polynomial feature expansion. Cellulase yield prediction was analysed using multiple regression models such as Ridge Regression, Bayesian Ridge Regression, Huber Regression, Partial Least Squares (PLS) Regression, Gradient Boosting, Extra Trees, XGBoost and ensemble models; and contamination risk was modelled using imbalance-aware classification with SMOTE and ensemble learning.

**Results:**

The Huber Regressor outperformed the other algorithms with an R^2^ of 0.9998, while PLS Regression performed well with an R^2^ of 0.9937, further validating the strength of the proposed feature representation. The soft voting ensemble classifier was 83.5% accurate and had an AUC-ROC of 0.9045 for the contamination prediction.

**Discussion:**

The integrated framework is a proof-of-concept showing the potential of simultaneously modelling productivity and contamination risks, which can aid in the evaluation of fermentation operating conditions in a risk-aware fashion. The data used in the benchmark is synthetic, so the predictive results are meant to show the validation of the proposed surrogate modelling framework, and not that the predictive ability of the framework is of industrial scale. The framework will be validated in the future with bigger fermentation datasets obtained from experiments and expanded to the real-world fermentation process optimization.

## 1 Introduction

In recent years, with the emergence of a need for petroleum alternatives, research on microbial bioprocesses for the manufacture of biofuels, enzymes and other bio-based chemicals has been gaining momentum (Ramchuran et al., 2023). Of these, cellulase enzymes are particularly important in breaking down lignocellulosic biomass to fermentable sugars, which are crucial for the biorefineries and sustainable biomanufacturing systems of the future (Ilić et al., 2023). Cellulase fermentation performance is still a research goal since several interacting operational factors such as temperature, pH, dissolved oxygen, agitation, inoculum size and feeding strategies have considerable impact on the yield of the products (Dey et al., 2022; Chai et al., 2022).

Although there have been great strides in metabolic engineering and synthetic biology, it has been difficult to accurately describe these non-linear relationships. Design of experiments (DoE) and mechanistic kinetic models are conventional optimization techniques that can be inflexible when using a large number of interacting variables simultaneously and can require a significant number of experiments (Zhou et al., 2021). Therefore, machine learning (ML) has become a supplementary data-driven approach that has the potential to approximate the complex fermentation behavior directly from the observed process variables (Campo-Manzanares et al., 2026). The ML models are not meant to stand in for mechanistic understanding but can offer efficient alternatives so that the rapid prediction, comparison of models and generation of hypotheses can be achieved.

Artificial neural network (ANN), support vector machine (SVM), ensemble learning and deep learning are found to be effective approaches to predict the performance of fermentation and optimize the operating conditions of fermentation (De Farias Silva et al., 2022; Vinitha et al., 2023; Aziz et al., 2020; Ge et al., 2023; Pan, 2023). These approaches have led to modeling of enzyme levels, substrate use and process behavior of nonlinear relationships; and, in many cases, have been superior to conventional statistical approaches (Campo-Manzanares et al., 2026; Srivastava and Atri, 2025; Khaleghi et al., 2021). Recently, the dynamic optimization of feeding strategies, the regulation of dissolved oxygen and other operational parameters in microbial fermentation systems by reinforcement learning and advanced control strategies have been explored (Pan, 2023). Concurrently, advances in Digital Twin technologies have emerged as a promising way to combine predictive models and interactive visualization and simulation environments to aid process analysis and scenario exploration (Herwig et al., 2021; Appl et al., 2020; Park et al., 2021).

The available literature, however, has been focused on maximizing yield with relatively less consideration given to a quantitative assessment of contamination risk and process robustness. Reduced productivity, high cost of operation, and unreliable fermentation process due to contamination are one of the major problems associated with fermentation and are often encountered (Pan, 2023; Wang et al., 2025; Hematillake et al., 2022; Wu et al., 2025). Similarly, there are a number of reports of optimizing yield while neglecting crucial considerations to ensure process stability and metabolic burden and operational resilience in up-scaling (Park et al., 2023; Wang et al., 2023; Mukasine et al., 2024; Yee et al., 2025; Han et al., 2020; Banerjee and Mukhopadhyay, 2023). Based on these observations, the productivity and process risk must be dealt with together while developing the predictive framework for fermentation analysis.

In this context, Digital Twin concepts are not just a validated representation of an industrial fermentation system but are computational environments that combine predictive models and interactive visualization. The current work is not intended to prove the industrial use or process control. Rather, it designs and tests a surrogate modelling methodology based on a public benchmark data set to explore the possibility of providing the cellulase yield and contamination risk prediction in a common analytical framework using multiple machine learning models. The benchmark data set contains 20 experimental observations of fermentation data and 980 synthetic data points which allows a systematic comparison of regression and classification methods under controlled conditions. The obtained model can incorporate domain information, use multiple regression techniques such as Partial Least Squares (PLS) regression and ensemble learning, and be contamination aware to address the imbalance problem, thus creating an interactive environment for benchmarking evaluation and exploratory decision support.

The three main contributions of this work are: To this end, a single surrogate modeling tool is first developed for predicting yield of cellulase and contamination risk at the same time based on variables of fermentation process. Second, multiple regression and classification models are systematically tested by taking a common pipeline of preprocessing and feature engineering and setting benchmark performance for various learning strategies. Thirdly, an interactive Digital Twin inspired interface is proposed to enable visualization of predictions and exploratory analysis in the benchmark environment. The framework is assessed with a benchmark dataset that consists of synthetic observations, and thus the predictive performance reported should be viewed as a means of methodological evaluation of the surrogate modeling approach and not as validated performance in industry. However, the proposed framework offers a replicable starting point to be expanded and tested with fermentation data collected from experiments in the future.

## 2 Methodology

### 2.1 Principle of the proposed tool: FERM-AI twin

There are several operational parameters (temperature, pH, dissolved oxygen, agitation, inoculum size, substrate feeding and culture time) that play a role in microbial cellulase fermentation and these parameters are involved in complex nonlinear interactions. All these factors affect enzyme productivity and stability of fermentation process, and disturbances like oxygen deficiency, adverse conditions and microbial contamination can significantly affect the fermentation towards production. Although modelling these relationships is still difficult due to the small number of fermentation datasets and the strong correlation of the process signals in these datasets. In these cases, generic statistical methods and only mechanistic models might not be easily applicable to a variety of operating situations.

To overcome these challenges, this study presents a risk-aware machine learning-based surrogate modeling framework for benchmark evaluation of cellulase fermentation processes called FERM-AI. FERM-AI is not a validated industrial Digital Twin or mechanistic simulation of biochemical kinetics, but rather a Statistical Digital Twin that can be developed using Supervised Machine Learning (SML) based on a benchmark dataset of fermentation operating conditions and outcomes. The framework can be used for more interpretable computation for assessing predictive models, investigating interactions of process variables, and comparative analysis of the operating conditions in fermentation processes.

The proposed structure comprises two complementary prediction tasks, and a single-fuzzy-framework analytical workflow to work them together. The first one attempts to estimate the cellulase yield from the controlled fermentation parameters from multiple regression algorithms. The second task assesses the contamination risk of the same process variables, but with classification models that are aware of the imbalances. By looking at productivity and contamination both at once, the framework can explore possible productivity-contamination compromises, that would not have been possible to do using only the yield prediction.

As shown in Figure 1, the proposed FERM-AI framework includes both a FERM layer and an AI layer. Overall architecture of the proposed FERM-AI framework is shown in Figure 1, which consists of two layers, namely, FERM layer and AI layer. The workflow starts with a benchmark fermentation data, followed by data preprocessing, and biologically meaningful representations of process optimality, stress and variable interactions are achieved through domain-informed feature engineering. The processed data are then fed into several multiple regression models to predict cellulase yields and ensemble classification models are produced to assess the contamination risk. The performance of the models is assessed according to the metrics of the tasks and the trained models are then embedded in an interactive visualization interface on the Web through which one can envision the possible prediction scenarios and visualize the results of the models. FERM-AI offers a modular and interpretable design to enable one to benchmark the machine learning methods used for fermentation analysis and set a baseline for future validation with the help of experimentally obtained fermentation data.

**FIGURE 1:**
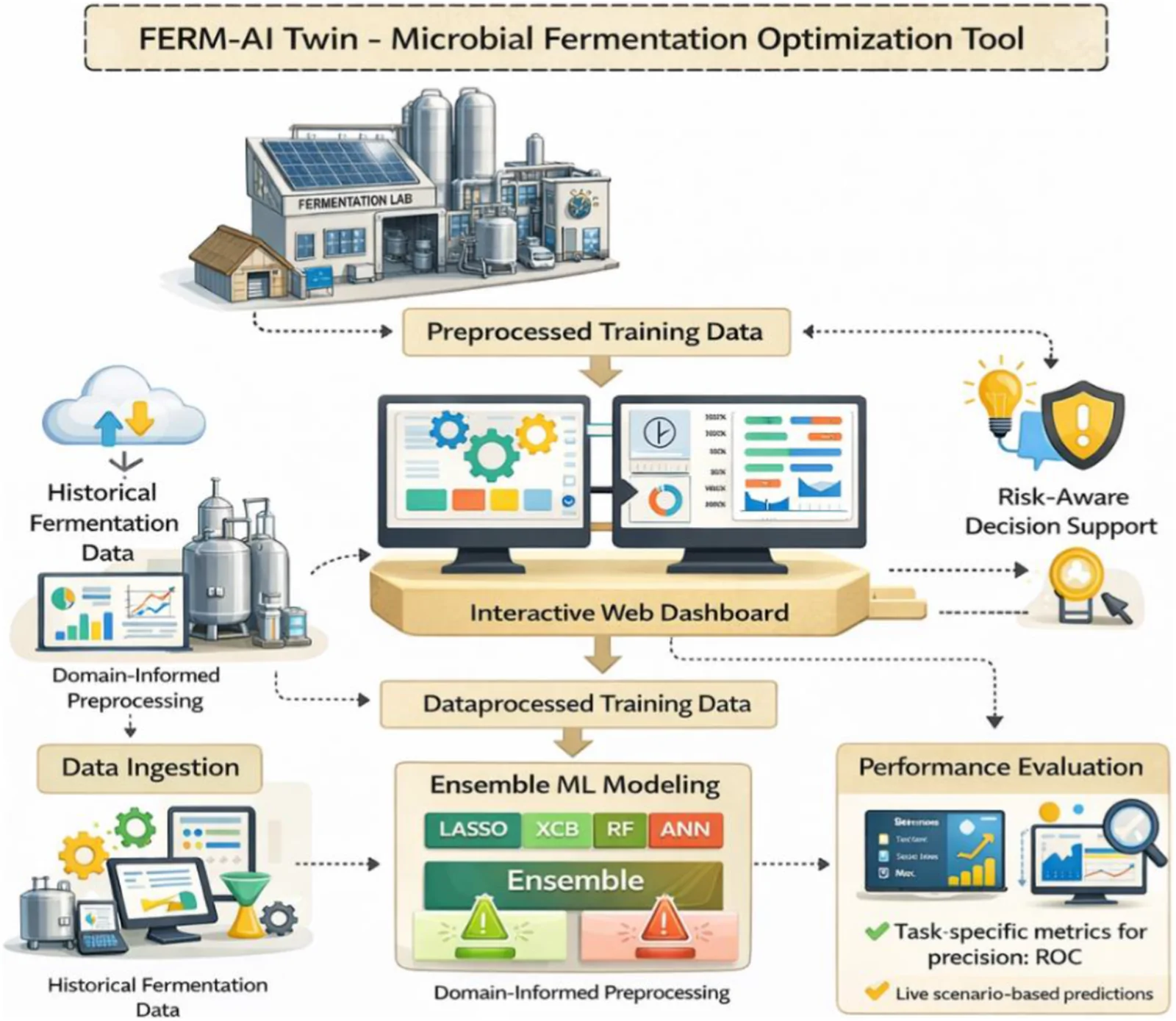
Proposed architecture of the FERM-AI Twin framework which shows data processing of fermentation process, modelling of the ensemble-based yield and contamination, and risk-sensitive decision support using an interactive dashboard.

### 2.2 Description of dataset and formulation of the problem

The present study uses a publicly available benchmark dataset which was uniquely developed to enable the development and evaluation of machine learning models used for optimizing cellulase fermentation (Konwar, 2024). The benchmark consists of 1,000 fermentation observations, of which 20 observations were taken from real experiments of cellulase fermentation in a laboratory bioreactor (5-L) and the other 980 observations were synthetically generated using data generation routines written in Python using Pandas and NumPy libraries. The main objective of the optimization was to maximize cellulase activity (Max FPU/mL) and this data set was developed to assist in predictive modeling of the cellulase production.

The fermentation records are summarizing the operating conditions for a given fermentation run, instead of continuous operating condition trajectories. The measured parameters are: average fermentation temp, average pH, minimum dissolved oxygen, size of the inoculum, maximum agitation speed, total amount of lactose added, total culture time, total amount of culture media, contamination status, notes of the operator, and cellulase activity. These variables form the input space to be used for feature engineering and predictive modelling. Table 1 summarizes the variables for the benefit of those who wish to cite them. All the benchmark variables, their units of measurement, ranges observed and their roles in modelling are summarized in Table 1 for those who need to refer to the variables.

**TABLE 1:** Description of fermentation dataset features including variable names, definitions, units, and identification of primary response variables.

Feature name	Category	Description	Unit	Observed range/Categories	Modeling role
Run_ID	Identifier	Unique fermentation batch identifier	–	TRICH_001–TRICH_1000	Identifier
Avg_temp	Process variable	Average fermentation temperature	°C	20.52–35.57	Input
Avg_pH	Process variable	Average fermentation broth pH	–	2.61–7.36	Input
Min_DO %	Process variable	Minimum dissolved oxygen	%	−37.29–86.01	Input
Inoculum size (mL)	Process variable	Inoculum volume	mL	34.46–159.70	Input
Max_RPM	Process variable	Maximum agitation speed	rpm	173.33–307.83	Input
Total feed of lactose (mL)	Process variable	Total lactose supplied	mL	−475.61–3126.96	Input
Total culture duration (days)	Process variable	Fermentation duration	days	2.93–8.40	Input
Total media volume (mL)	Process variable	Working culture volume	mL	2,344.44–5,796.34	Input
Contamination (Y/N)	Categorical	Contamination status	Yes/No	N = 573, Y = 427	Classification target (reconstructed during preprocessing)
Notes	Qualitative	Operator observations	Text	Low/Medium/High	Auxiliary input
Max FPU/mL	Response variable	Maximum cellulase activity	FPU/mL	−4.843–9.076	Regression target (reconstructed during preprocessing)

An exploratory analysis of the raw data was carried out as a *post hoc* analysis of the benchmark in order to better understand the statistical characteristics of the benchmark. This analysis showed that all of the eight main process variables had close to Gaussian distributions with very slight skewness, and were poorly correlated (Pearson’s correlation |r| max ∼0.045). In addition, there were several observations that were physically unrealistic such as negative dissolved oxygen (DO) readings, negative lactose feed volume and negative measurements of cellulase activity. These observations agree well with independently sampled synthetic benchmark data to extend the operational design space, instead of the experimental coupled trajectories of fermentation. As a result, the benchmark contains fermentation runs that have been obtained from experiments, but should mainly be viewed as a methodological benchmark data set for the development of machine-learning models and not as an extensive experimental fermentation data set.

The FERM-AI preprocessing pipeline also preprocesses both the prediction targets before model training, and reconstructs the prediction targets using the synthetic expansion performed by the dataset authors. Specifically, the original cellulase activity values are replaced by an artificial value based on a weighted combination of the optimality of the process, the productivity of the substrate, the availability of dissolved oxygen, the use of lactose, the length of the fermentation period, and cumulative stress to the process, with controlled Gaussian noise applied to maintain stochastic variation. Similarly, contamination labels are determined probabilistically using a risk model, which incorporates temperature, dissolved oxygen, pH stress and culture time, and then thresholded to binary labels. This controlled signal construction allows for iterative testing of the proposed surrogate modeling approach in a repeatable environment with meaningful bio-constructed relationships between the engineered features. Information about the full target reconstruction method, mathematical formulation and implementation are given in the Supplementary Synthetic Data Description.

The resulting benchmark is two supervised learning tasks that are complementary. The main objective is a regression problem of predicting the maximum cellulase activity (Max FPU/mL) and the secondary objective is a binary classification problem to predict the contamination. After target reconstruction, the contamination labels showed a moderate class imbalance (573 non-contaminated and 427 contaminated observations) and during model training, the Synthetic Minority Over-sampling Technique (SMOTE) was used. For the purpose of information leakage prevention, SMOTE was used only after train-test split but before the development of the classifiers whereas the independent test set was not used for any SMOTE operation during the whole model evaluation process.

The benchmark has a restricted number of operational variables but domain-informed feature engineering was carried out to add more activity aspects of the fermentation. The total number of engineered features was 36 including 44 predictive features were used for model development. These engineered descriptors represent measures of optimality of the process, environmental stress, productivity of the substrates, nonlinear interactions, process ratios, deviation measures, and composite biological indicators.

These data lead to the formulation of cellulase fermentation analysis as a dual-task supervised learning problem: regression models are used to estimate cellulase productivity, while classification models are based on the same set of operating variables that are used to estimate contamination risk. Such a combination of complementary prediction tasks allows for the simultaneous evaluation of the level of productivity and operational robustness in the same analytical process.

Lastly, the benchmark does not consider dynamic fermentation trajectories or consider process metadata of fermentation process, like fermentation mode (batch, fed-batch, continuous), feeding schedules, substrate consumption kinetic, oxygen uptake rate, carbon evolution rate or reactor control strategy. It is therefore recognized that every observation of a fermentation is an operational state of a process described by joining together some state descriptors of the process, but not a full temporal bioprocess trajectory. In this context, FERM-AI is introduced as a readily replicable surrogate modeling framework for comparison of machine learning methods for fermentation predictive and interactive scenario analysis. The reported predictive performance should therefore be considered as a validation of the proposed methodological framework, in the benchmark environment, and not as an evidence of the predictive capability at an industrial level.

### 2.3 Data preprocessing and feature engineering

The benchmark dataset was first standardized prior to model development, for consistency between regression and classification tasks. The missing data was checked on the continuous process variables and missing values were filled with the mean value of the feature (arithmetic mean) to maintain the complete data set. Categorical labels were converted to numerical (binary) representation for supervised classification. All continuous input features were then standardized with the StandardScaler to ensure that the distributions of the features are the same (zero mean and unit variance) before training the model, to remove the differences in magnitude of the features, and to make the optimization more stable. These pre-processing steps help to reduce the numerical instability, but fermentation data often contain low predictive signals due to the complex nonlinear interactions between the operating variables, biological variability, and availability of the experimentally measured observations. As a result, domain-informed feature engineering was used to create surrogate descriptors that are biologically plausible, yet also capture patterns within the benchmark data that can be informative and used to boost the overall quality of the informative patterns.

The original fermentation variables were reduced to 36 engineered features which gave rise to 44 features for use in model development. These engineered variables were summarized in Table 2 as ten categories of variables that describe the functional response of the process to the variables. Categories include process optimality, environmental stress, substrate productivity, interaction effects, normalized process ratios, nonlinear transformations, deviation measures, Boolean operating-state indicators, logarithmic transformations, and composite biological descriptors. They are used together to give complementary information, which cannot be obtained from the raw process variables alone.

**TABLE 2:** Engineered feature sets and their functional contributions in signal improvement, biological understanding and model performance.

Feature category	Number of features	Purpose
Optimality features	5	Quantify proximity to biologically optimal fermentation conditions
Stress features	3	Capture deviations from favorable operating conditions
Productivity features	3	Estimate substrate utilization and productivity potential
Interaction features	5	Model pairwise interactions among process variables
Ratio features	7	Normalize operating variables and improve comparability
Polynomial features	3	Capture nonlinear relationships between fermentation parameters
Deviation features	2	Quantify departures from reference operating conditions
Boolean flag features	3	Encode threshold-based operating states
Logarithmic features	3	Stabilize feature distributions through logarithmic transformation
Composite feature	1	Aggregate multiple stress indicators into a unified stress index
Total engineered features	36	Domain-informed descriptors derived from the raw process variables

Complementary stress features were developed to measure the deviations from good operating conditions. These are not direct measures of productivity, but are a measure of accumulated environmental stress during fermentation. Individual stress measures were then summated to create a composite Total Stress Index which allowed the learning algorithms to model the combined effect of multiple suboptimal operating conditions in a single, biologically interpretable stress measure. To represent the efficiency of fermentation, productivity features were generated by normalizing the substrate availability, reactor loading and process duration to produce normalized productivity features. These variables are used as an indirect measure of the efficiency of use of the substrate as well as the enzyme productivity without having to explicitly model biochemical reaction rates.

Several interaction features were added to account for the coupled effects of the different environmental variables of interest, as the fermentation performance is highly dependent on these. They can be either interaction between variables such as temperature and pH, or between other variables such as dissolved oxygen and agitation speed or between substrate loading and fermentation duration. These interaction terms allow for the modelling of nonlinear dependencies which would otherwise need much more complex model architectures, and allow traditional machine learning algorithms to approximate such dependencies.

Other engineered features are ratios that normalise the operational quantities for easier comparison between fermentation runs, polynomial features that encode higher-order nonlinear relationships between selected quantities, deviation features that quantify deviations from reference operating conditions and Boolean indicator features that encode biologically meaningful threshold conditions; and logarithmic transformations that reduce skewness and stabilise feature distributions for the subsequent model training. All engineered features are mathematically defined, equations are provided for implementing the features, variable descriptions are included, and biological motivations are given, in the Supplementary Feature Engineering Documentation. These engineered variables were generated to enhance the representation learning in the benchmark dataset, based on domain knowledge, and in that sense are not mechanistic biomarkers *per se*, but rather surrogate markers.

It is important to note that the feature space used in the engineered creation was for the purpose of benchmarking the evaluation of predictive modeling methodologies. The engineered variables are therefore meant to enhance the weak predictive signals in the benchmark dataset and to enable the experimental comparison of machine learning models in a controlled setting, with the synthetic observations. The synthetic observations and reconstructed prediction targets are part of the benchmark dataset and the engineered variables are meant to enhance weak prediction signals in the benchmark dataset and to enable the controlled experimental comparison of machine learning models. The predictive performance reported in this study is thus representative of the performance in the benchmark environment and not necessarily a reflection of the behavior of the fermentation at industrial scale and therefore should not be interpreted as such. These engineered descriptors will need to be further validated with fermentation data obtained from experiments to determine the generalizability of these descriptors under real bioprocess conditions.

### 2.4 Machine learning models and training strategy

A wide range of machine learning algorithms were tested for modeling of the prediction of cellulase yield and detection of contamination risk in weak-signal fermentation. The models chosen were regularized linear methods, robust regression methods, ensembles of decision trees and ensembles of classifiers based on probabilities. This diversity was designed to evaluate complementary learning behaviors, the robustness when the engineered features are correlated, and to minimize overfitting in partially synthetic datasets from fermentation processes, which have a relatively small number of data points.

#### 2.4.1 Yield prediction models

Cellulase yield was predicted using several different regression algorithms of different complexity and regularization. Engineered interaction features were a potential source of multicollinearity among the predictors, so ridge regression and Bayesian ridge regression were added to the regularized linear baselines. Bayesian Ridge also gave the possibility of a small-scale dataset to normalize by a probabilistic regularization. To enhance robustness against experimental and synthetic observations of fermentation data that often suffer from noise and possible outliers, Huber Regression was used. The technique is sensitive to small residuals, yet it has decreased influence from extreme deviations. Gradient Boosting Regression, Extra Trees Regression and XGBoost Regression were added to identify nonlinear relationships and higher order fermentation process interactions between operational process variables. The ensemble based methods were chosen because they have been widely used in structured tabular prediction tasks.

Partial Least Squares (PLS) Regression was also included as a representative regression model because it is widely used in chemometrics, in other fermentation process monitoring and multivariate bioprocess analysis, especially in the case of small sample size and correlated process variables. PLS differs from standard linear regression because it transforms the original predictor space onto a smaller space of so-called “latent components” that maximize the covariance between the predictor variables and the response, which helps to make the model more robust towards the presence of multicollinearity caused by feature engineering through the domains. The number of latent components was optimised by cross-validation on the training set and the set that gave the best performance was selected for further evaluation of the model. The introduction of PLS Regression will give a sound multivariate framework to which the proposed robust and ensemble learning techniques can systematically be compared.

Several techniques for taking advantage of the complementary predictive power of several base regressors and to boost overall robustness were evaluated, such as Meta-learning techniques (Stacking Regression, Weighted Blended Ensembles). Systematic evaluation of complementary predictive strategies was possible using the datasets related to weak-signals fermentation, with correlated engineered features and limited experimental information, as an example, by comparative assessment of regularized linear models, latent-variable regression (PLS), robust regression and nonlinear ensemble learning.

#### 2.4.2 Contamination risk models

To optimize the interpretability, robustness and sensitivity to rare events of process failures, multiple classification algorithms were assessed for contamination-risk-prediction. Logistic Regression was added to the list of interpretable probabilistic baseline classifiers that could be used to generate calibrated contamination-risk estimates. Random Forest, Gradient Boosting and XGBoost classifiers were chosen to capture the nonlinear interactions and various operational patterns linked to contamination events. A Soft Voting Ensemble was used to fuse the probabilistic output of several classifiers, to enhance robustness and minimize dependency on classifier behaviour. Contamination detection was considered a risk sensitive task so in addition to predictive accuracy a focus was placed on the recall, F1-score and AUC-ROC performance. The resultant classification framework can be used to provide interpretable and risk-informed monitoring of the process conditions during fermentation in the proposed surrogate modeling environment.

#### 2.4.3 Training strategy and evaluation

The yield target variable was log-shifted before being used for regression training to gain learning stability by decreasing its skewness and stabilizing variance. Training-test partitioning and SelectKBest pre-processing were used to assess feature relevance, which enabled informative engineered features to remain while diminishing the impact of weak features.

To prevent information leakage between the training and test sets, a stratified train-test split was done before feature scaling, feature selection, polynomial expansion and model fitting. Only the training data was used to fit the parameters of the standardisation and the resulting parameters were used to process the held-out test data. Likewise, the feature-selection and polynomial-transformation techniques were learned from the training partition, and then applied to the tests partition.

Cross-validation was employed for comparative model evaluation, ensemble weighting, and selection of the optimal number of latent components for the PLS Regression modelPerformance for regression models was measured by the coefficient of determination (R^2^), the root mean square error (RMSE) and the mean absolute error (MAE), and the accuracy, precision, recall, F1-score and AUC-ROC were used to measure the performance of the classification models. The reported performance metrics are indicative of simulated fermentation performance with features added to the simulated signals, so are not directly applicable to industrial fermentations but instead relate to a controlled performance simulation set, which mimic fermentation conditions with features added to the simulated signals.

### 2.5 Class imbalance handling and risk-aware modeling

Class imbalance is a critical factor to take into account when developing classifiers, as contamination of fermentation processes is relatively rare but critical to the process. After the reconstruction of the targets, there was a total of 573 non contaminant observations and 427 contaminant observations, corresponding to 57.3% and 42.7% respectively in the benchmark dataset. This imbalance is not extreme, but classifiers induced directly from the original distribution can be biased towards the majority class and have a high overall accuracy at the same time, causing them to have a lower sensitivity to the contamination events. The proposed classification framework aimed to enhance minority-class detection for the following reason. If a batch becomes contaminated, it may result in the entire batch being thrown away and a significant loss of production.

To overcome this problem, the Synthetic Minority Over-sampling Technique (SMOTE) was used in the contamination-risk modelling. Whereas SMOTE generates synthetic minority class samples by interpolating between neighboring contaminated samples in feature space instead of simply copying the samples. This will help the classifiers learn more robust decision boundaries for detecting contamination events while increasing the probability of contamination events in the training set but maintaining local feature relationships.

The SMOTE was only used in the training partition after train-test splitting and before training the classifier, to avoid information leakage. The classification results were not computed by oversampling the validation data or test set, nor did they rely on any manipulation or transformation of the data distribution to yield the results. In addition, SMOTE was only applied to the contamination classification task and not during the training of the regression models for prediction of cellulase yield, as the continuous response variable was used. This separation allows to maintain the natural distribution of the regression target variable, but only generates synthetic samples according to the imbalance correction needed for binary classification.

To address the dual objective design of the proposed FERM-AI framework, the selective application of SMOTE is considered. The selective application of SMOTE is considered due to the dual objective design of the proposed FERM-AI framework. The framework does not assume that optimizing the fermentation is just a productivity prediction problem, but also separately models the amount of enzymes produced and contamination risk, enabling the analysis of productivity as well as the reliability of operation. The strategy allows the proposed surrogate modelling framework to consider potential trade-offs between cellulase production, and risk of contamination, within the benchmark environment, rather than just assessing these conditions based on yield prediction.

### 2.6 Model evaluation and performance assessment

Metrics for evaluating model performance were different for each task (regression, classification) and each learning objective. The proposed framework tackles two complementary prediction problems: (1) the prediction of yield of cellulase and (2) the classification of contamination risk, therefore task-specific performance measures were used to evaluate predictive accuracy allowing a fair comparison between the various machine learning methods. All reported figures are in the context of the benchmark data set and should, therefore, be seen not as predictive measures for industry but as a measure of methodological performance.

Three complementary performance parameters were used for the regression job in which the maximum cellulase activity (Max FPU/mL) was predicted. Overall measure of goodness-of-fit was used as coefficient of determination (R^2^) was used to quantify the proportion of variance in the response variable explained by the model. The Root Mean Square Error (RMSE) calculated the magnitude of the prediction errors with higher weights for the larger errors, whereas the Mean Absolute Error (MAE) was a good estimate of the average prediction error that is less affected by extreme observations. These metrics collectively provide a holistic evaluation of the accuracy of regression and consistency of the predictions.

The accuracy, precision, recall, F1-score and area under the receiver operating characteristic curve (AUC–ROC) were used to assess the performance of the models when predicting contamination. While the overall accuracy of the classification shows the overall performance of the prediction, the introduction of precision and recall was to measure the balance between false positive and false negative predictions. In the context of the benchmark framework, it is particularly interesting to examine recall, as this indicates the capability of the classifier to detect contamination events, and to examine the F1-score as this combines the precision and recall. The AUC–ROC was also employed to verify the discrimination ability of the classifier for different decision thresholds, which is threshold independent.

All the evaluation metrics were computed on an independent held-out test set, which had no overlap with the model development. Stratified train-test splitting was used to maintain the contamination class distribution of the data sets, and the preprocessing operations (feature scaling, feature selection, polynomial feature generation, and SMOTE-based oversampling) were only performed on the training partition, and then applied to the test partition. The evaluation approach reduced the leakage of information and guaranteed that the results reported were unbiased estimates of the performance of the benchmark given the same experimental conditions.

Based on the obtained performance metrics, the different regression, classification and ensemble learning techniques were then compared in the proposed FERM-AI surrogate modelling framework. The quantitative results in Section 3 should therefore be seen as proof of the usefulness of the framework to evaluate the benchmark, and to compare different methodologies, but not as a direct basis for estimating predictive performance for use in an industrial fermentation system.

### 2.7 Explainability and interpretability

Interpretability is a critical aspect of data-driven fermentation analysis as just performing well is not enough to offer any understanding of what operational factors are important to cellulase productivity or risk of contamination. Based on this, the proposed FERM-AI framework includes an explainability component to systematically analyze how well the ML models explain the results based on the raw process variables and engineered features. The framework is not designed to be a predictive system, but instead to only identify those process characteristics most closely linked with benchmark yield and contamination effects, to provide increased transparency and reproducibility of the model.

The relative importance of the individual variables was calculated for the ensemble-based learning algorithms using feature importance analysis. Both engineered features (based on the domain) and the original fermentation parameters (such as temperature, pH, dissolved oxygen, agitation speed, inoculum size, lactose feed and fermentation duration) are included in the analysis. Many of the engineered descriptors are transformations of the raw process variables and their importance on both levels is useful to understand the relationships between the process variables that are captured by the models, rather than just the raw variables.

Special attention is given to the engineered descriptors of process optimality, environmental stress and interaction effects. Each of the optimality features measures the closeness of the operating conditions to the empirical optimum fermentation regimes using a normalized optimality function, while the stress features represents the cumulative deviation of the operating conditions from the optimum regimes. The nonlinear interactions between the key fermentation parameters (such as temperature–pH coupling, dissolved oxygen (DO) – agitation interaction, and effects of substrate utilization) are captured by interaction features. All of these mathematically defined surrogate descriptors allow the learning algorithms to be easily interpretable, while capturing the behavior of the processes. All engineered features are mathematically formulated and the steps to implement them are described in the Supplementary Feature Engineering Documentation.

In the context of yield prediction, feature importance analysis is used to reveal the effect of the variables and engineered descriptors on the yield of the cellulase, which helps to point out operating conditions associated with their desirable performance. The same applies for contamination risk prediction: In this case the feature importance gives some information about the combinations of environmental conditions with the highest likelihood of contamination. The analysis of these complementary prediction tasks can be done individually, providing the opportunity to interpret possible operating conditions compromises between productivity and contamination risk in the benchmark environment.

The feature importance results from the proposed framework represent explanations of the learned surrogate relationships within the benchmark and should not be interpreted as experimentally validated microbial physiology indicators due to the use of a synthetic data set for evaluation. However, the explainability framework offers a clear and transparent way to compare machine learning models, interpret the influence of engineered representations of a problem space, and reproduce the interpretation of benchmark predictions. To prove the consistency of the found relationships between the identified features under real bioprocess conditions, future validation with datasets acquired experimentally from fermentation will be necessary.

### 2.8 Dashboard implementation

In order to operationalize the proposed FERM-AI Twin framework and provide an interactive decision support, a web-based dashboard called Cellulase Fermentation Surrogate framework was created. The dashboard is the interface where the user interacts with the system and incorporates the ingestion of data, model execution, visualization of results, and analysis of scenarios in a single interfaces platform. The interface is tailored to accommodate process engineers and the researchers in pursuing discovery of the behavior of fermentation and assessing the optimization strategies in a transparent and intuitive way.

The dashboard is divided into several functional tabs, which are linked to a particular phase of the modeling and decision-support process:

- This Home tab serves as a window into the system and it gives an option to upload fermentation datasets in spreadsheet format. The users are able to upload the excel files with fermentation parameters and results which are in turn validated and set to be analyzed. The data underpinning of all subsequent modeling and simulation activities is determined by this tab and guarantees the ability to apply the system to new fermentation data without having to manually reconfigure it.
- The Overview tab gives the general overview of the dataset uploaded and the modeling framework. In this section, the contextual information about the fermentation process, the range of the surrogate framework, and the capabilities of the system in terms of analytics are provided. It can be used to get oriented users prior to starting the model training or interpretation especially those who are first-time users.
- The Train Models tab makes it possible to engage the machine learning pipeline to the dataset that is uploaded. In this tab, cellulase yield prediction ensemble regression models and contamination risk detection classification models are being trained with the preprocessing strategy and feature engineering strategy described above. The tab enables users to launch training processes and optimizes the models towards future evaluation and implementation in the surrogate framework environment.
- The Results tab presents the quantitative activity of trained models with the help of task-sensitive evaluation metrics. Regression measures are provided to yield prediction including R2, RMSE, and MAE, whereas to measure the performance of contamination prediction, accuracy, precision, recall, F1-score, and AUC-ROC are provided. Comparative evaluation of various models and ensembles can be done by means of this tab and users can assess the predictive reliability of various models before using them to operational conditions.
- The main decision-support part of the dashboard is represented by the Surrogate framework tab. In this section, the user is allowed to input the operating conditions of fermentation and receive the related predictions of maximum cellulase activity and the risk of contamination. The users can apply parameters of the process to simulate alternative operating scenarios and see how they are expected to affect productivity and process stability. This interactive capability represents the principle of the surrogate framework since it allows virtual experimentation and does not require physical bioreactor testing.

## 3 Results

### 3.1 Dataset preprocessing results

This study used a benchmark dataset of 1,000 fermentation observations, with 20 of these observations obtained by experiments and 980 of these observations were synthetically generated observations provided by the original benchmark repository. The data was then divided into 800 training samples and 200 independent testing samples via the stratified train-test splitting. The test includes a vast variety of operating conditions such as temperature, pH, dissolved oxygen, speed of agitation, feeding of substrates, amount of inoculum and fermentation time, ensuring a controlled environment to test the machine learning models in various simulated operating conditions.

The following preprocessing was performed: Mean imputation for missing continuous variables, standardization of the continuous variables with the StandardScaler, feature selection, polynomial feature expansion and reconstruction of the regression and classification targets as described in Section 2.2. The preprocessing steps included in the benchmark were not designed to mimic experimentally observed fermentation behavior, but to create a more numerically stable, reproducible benchmark to compare machine learning models.

### 3.2 Feature engineering results

The benchmark dataset showed low correlations between the raw process variables, so biologically meaningful representations of fermentation behavior were enhanced using domain knowledge through feature engineering. Aside from the original operational descriptors, some additional engineered features were added, and the optimal process was determined based on these. The added features were used to capture process optimality, environmental stress, nonlinear interactions, and normalized process characteristics that are difficult to capture using individual features alone.

Feature selection and interaction-expansion strategies were used to enhance the stability of learning in the weak-signal data set, starting from a combination of raw and derived operational variables. A total of 10 most informative features were retained in the preprocessing pipeline, and polynomial interaction expansion produced an expanded feature space consisting of approximately 55 interaction-enhanced representations derived from the original and engineered variables. These created features allowed the learning models to learn nonlinear relationships and coupled interactions between the important fermentation variables, such as temperature, pH, dissolved oxygen, agitation rate, inoculum size, feeding of the substrate, and culture duration.

Engineered features were further investigated by feature-importance analysis with ensemble-based models for their contribution to the predictive behavior. Several features, engineered to incorporate interaction and optimality aspects, have been found to be more important than many of the raw operational parameters as shown in Figure 2. Specifically, signal-based representations that were stress-aware and interaction-based made a significant contribution to yield prediction, indicating that the models were better able to capture the complex fermentation trends in the dataset based on the signal processing, due to its domain guidance. The results are consistent with the possibility of using engineered representations to increase the interpretability and predictive consistency in the weak signal bioprocess data.

**FIGURE 2:**
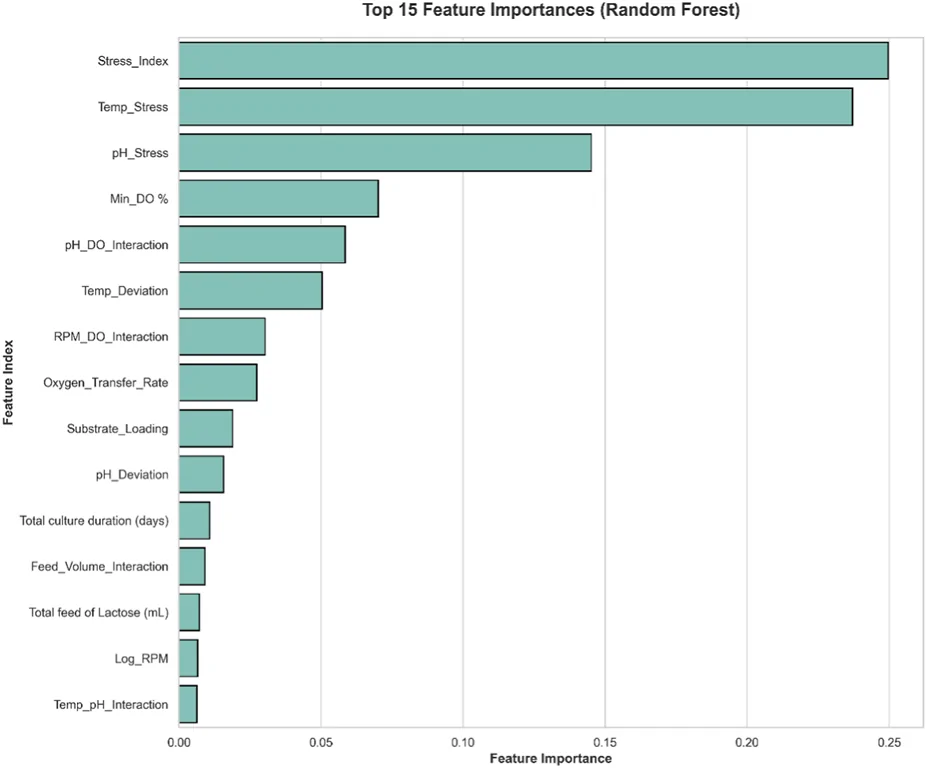
Random Forest feature importance ranking which indicates that engineered stress, interactions features are more important in predicting yield compared to raw process variables.

The engineered feature categories summarized in Table 2 were designed to strengthen weak predictive signals by incorporating biologically motivated representations of process optimality, environmental stress, and nonlinear interactions. The complete mathematical definitions of these engineered features are provided in Section 2.3 and the Supplementary Feature Engineering documentation.

The risk of overfitting that comes with both the high dimensionality of the interaction expansion, and the complexity of the resulting feature set was reduced by using SelectKBest feature selection, which only selects the most informative engineered representations before the model is trained. This dimensionality reduction step was performed to reduce the feature space while retaining signals that are important for fermentation behavior and are aware of interaction.

### 3.3 Class imbalance analysis and SMOTE results

After target reconstruction the contamination distribution was moderately imbalanced with 427 contaminated observations (42.7%) and 573 non-contaminated observations (57.3%). This imbalance can give a supervised classifer a tendency to classify most of its pattern as a majority class, which in turn can lead to decreased sensitivity to contamination pattern events, although not as severe as most (but not all) datasets in the field of industrial fault-detection.

To deal with this problem, SMOTE-based oversampling was applied exclusively to the training partition of the contamination classification task following train-test splitting, thereby preventing information leakage into the independent test set. Only the training subset underwent oversampling so that information could not percolate to the test set. After SMOTE, the effective training size used to contemplate contamination model grew to 1,006 samples which gave about balanced representation of both contaminated and non-contaminated classes. This equal distribution of training allowed the classifiers to acquire more discriminative decision boundaries in the detection of contamination.

The analysis of SMOTE showed that the contamination prediction performance is improved, specifically in the performance of recall and AUC-ROC, which are essential indicators of detecting rare failures in the processes. As Figure 3 demonstrates, ensemble classifiers and tree classifiers attained very high accuracy and discrimination and the baseline logistic regression did not, which means that imbalance correction was necessary in order to make meaningful risk modeling.

**FIGURE 3:**
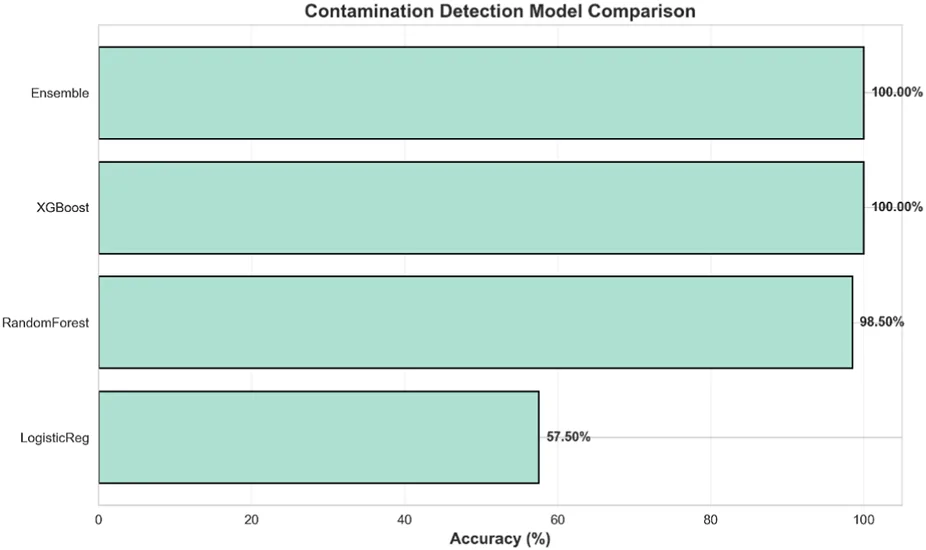
Comparison of the contamination detection models post-SMOTE, which perform better than logistic regression in terms of accuracy by an ensemble and tree-based classifier.

No oversampling was done to train a regression model and the resulting reconstructed productivity values were therefore preserved in their natural distribution. The limitation of the SMOTE algorithm to the contamination classification task ensured that the imbalance correction was done only when necessary to this task and was applied following a common evaluation procedure across the benchmark tasks.

### 3.4 Yield prediction models performance

A range of multiple regression algorithms (regularized linear models, latent-variable regression, robust regression, tree-based ensembles, meta-learning algorithms) were tested for the prediction of cellulase yield in the benchmark dataset. Performance of models was evaluated by the coefficient of determination (R^2^), Root Mean Square Error (RMSE) and mean absolute error (MAE) that facilitated systematic comparison of their predictive accuracy and generalization in complementary regression approaches.

A baseline model that simply predicts the mean of the observed data was also used to evaluate the models for comparison. This baseline is a prediction of the average yield of cellulase for all test samples regardless of fermentation conditions and thus offers little in the way of explanatory power. This baseline was significantly outperformed by all machine learning models showing that the proposed framework was able to learn relationships that extend beyond the average yield estimate.

The Huber regression model showed the best predictive agreement in the benchmark dataset with R^2^ = 0.9998 in the test set, RMSE = 0.0085 FPU/mL and MAE = 0.0050 FPU/mL. The predicted and observed yield values showed good agreement in all the samples as shown in Figure 4. The exceptionally high predictive agreement should be interpreted within the context of the benchmark dataset. Because the benchmark incorporates synthetically generated observations, reconstructed target variables, and domain-informed engineered features, the reported R^2^ reflects the framework’s ability to recover structured benchmark relationships rather than its expected predictive performance on experimentally acquired industrial fermentation data.

**FIGURE 4:**
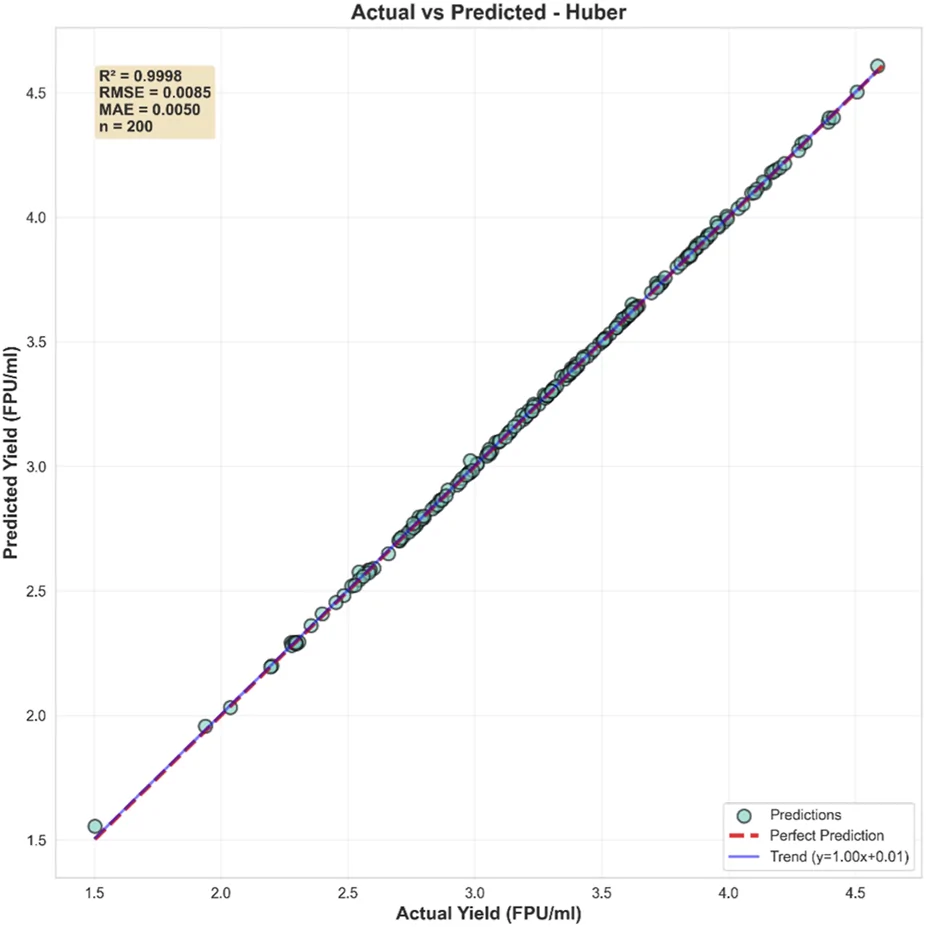
Relationship between experimentally represented benchmark yields and Huber regression predictions, demonstrating strong predictive agreement within the structured simulation-oriented dataset.

A highly competent performance was also shown by Bayesian Ridge regression, as test R2 was 0.9997, which proves that regularized linear models work well with appropriate feature engineering and target transformation to predict yields. Conversely, ensemble models that were tree-based were characterised with relatively low predictive accuracy. Extra Trees and Gradient Boosting had test R2 of 0.9163 and 0.8864, respectively, suggesting that either method is less prone to generalization when dealing with weak-signals.

Partial Least Squares (PLS) Regression was newly introduced based on recommendations of the reviewers and resulted in good predictive performance with R^2^ = 0.9937 and RMSE = 0.0449 FPU/mL, and MAE = 0.0310 FPU/mL. The strong predictive powers of the model while handling the multicollinearity caused by the domain-informed feature engineering was possible through PLS. The number of latent components were determined by cross-validation on the training set and 10 latent components were found to be the optimal number (Figure 5). The results showed that the performance of latent variable regression was inferior to Huber and Bayesian Ridge Regression, but the results were still consistent with the fact that the latent-variable regression is a good benchmark for fermentation data with correlated process variables and engineered surrogate variables.

**FIGURE 5:**
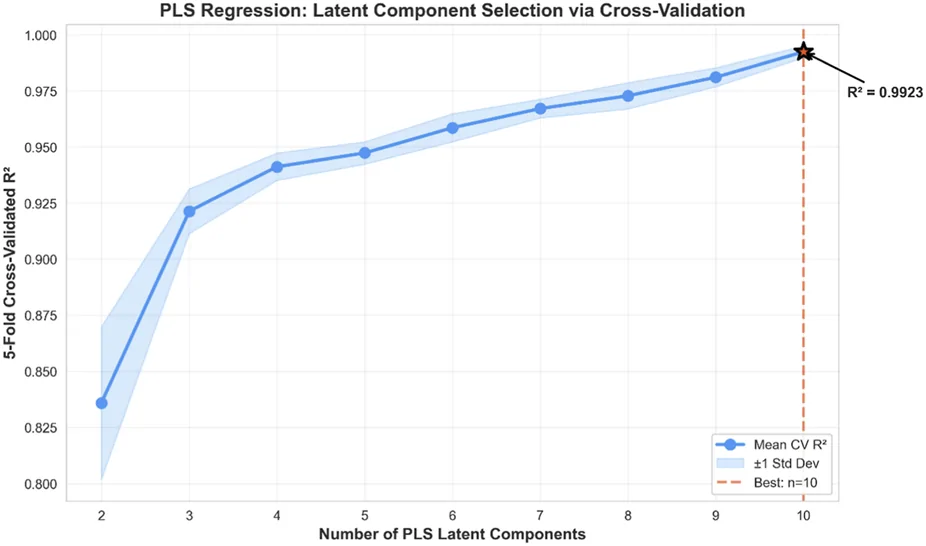
Cross-validation performance of Partial Least Squares (PLS) Regression as a function of the number of latent components. The highest cross-validated R^2^ was achieved with 10 latent components, which were subsequently used for benchmark evaluation.

The XGBoost model, however, was much more regularized, and yielded much poorer predictive performance (R2 = 0.6074). This behavior is likely attributable to the highly structured engineered feature space, where many nonlinear relationships have already been incorporated through biologically motivated transformations. Under these conditions, robust linear models require less model complexity than tree-based ensemble methods to recover the underlying benchmark relationships. Furthermore, the strong regularisation parameters used to XGBoost might have muted weaker interaction signals that are in the comparatively smaller and weaker signal dataset. The observations suggest that the structure of the feature space and the level of regularization are sensitive parameters of complex ensemble techniques for simulation-oriented fermentation benchmarks. Even though both stacking and blended ensemble methods yielded better robustness compared with single tree-based methods, neither of these two methods had a better predictivity than the Huber regressor as seen in Figure 6.

**FIGURE 6:**
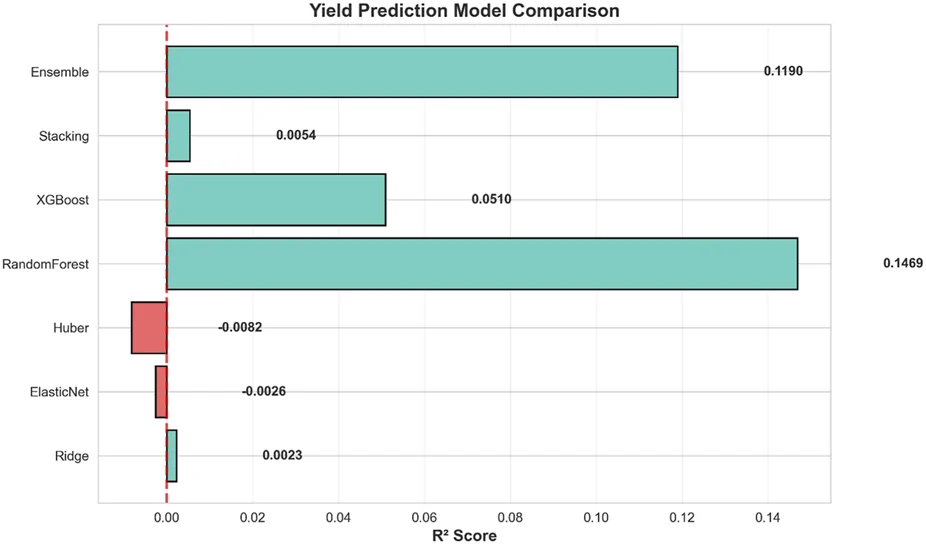
Comparative R^2^ scores of regression models evaluated on the benchmark dataset, highlighting differences in predictive consistency across linear and ensemble-based approaches.

The Huber model is also reliable as evidenced by residual diagnostics. Figures 7, 8 show that the residuals are closely clustered around the value of zero, there exist approximate normality and there is no trend, predicted or in fact observed yield values.

**FIGURE 7:**
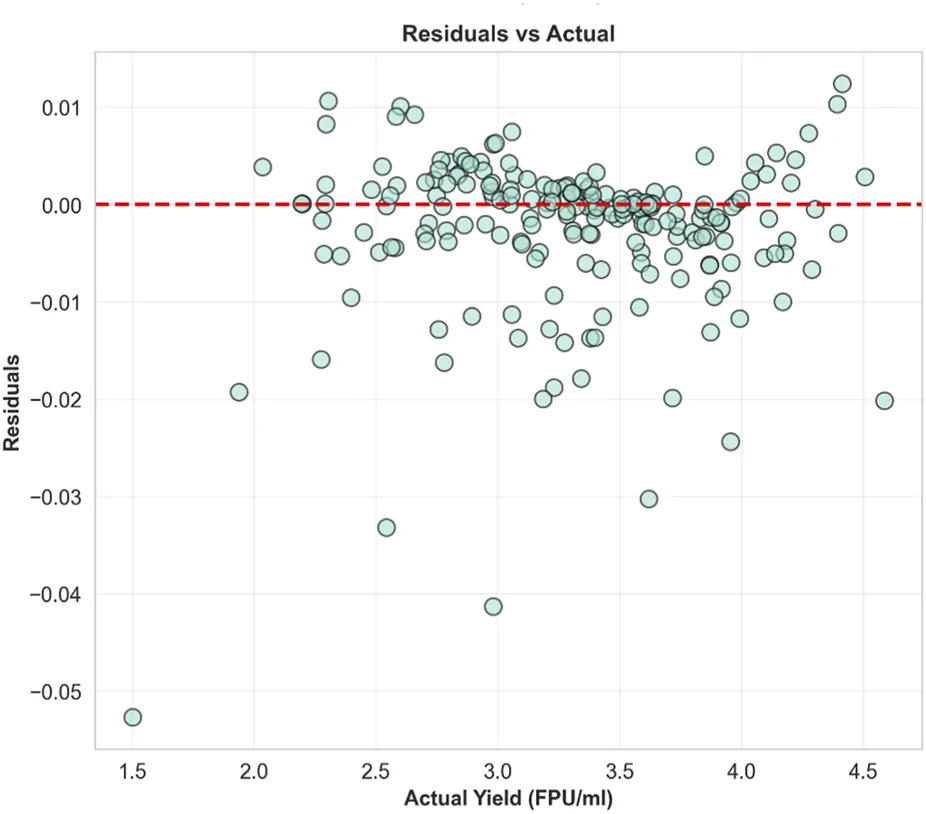
Residual diagnostics for the Huber regression model showing residual concentration near zero with limited systematic deviation across the evaluated benchmark samples.

**FIGURE 8:**
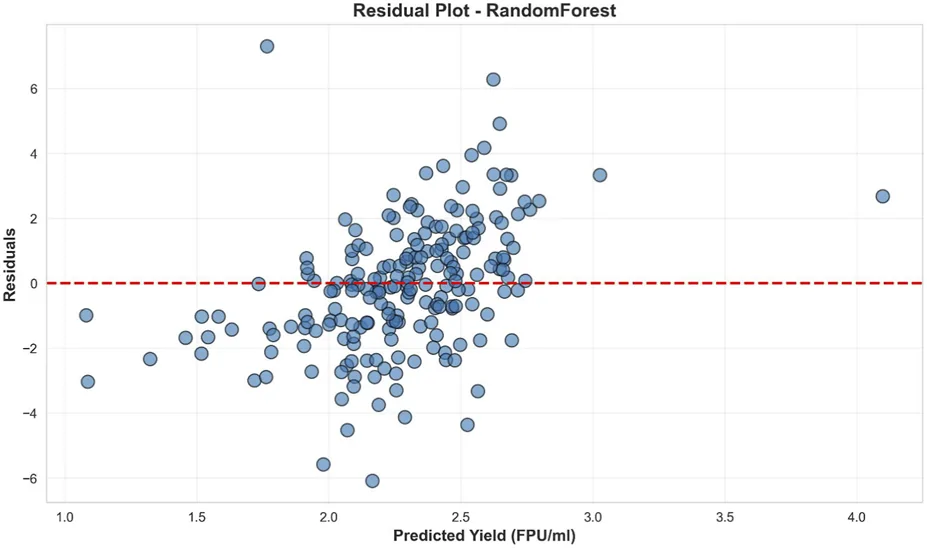
Residual distribution of the Random Forest model showing greater dispersion and variability compared to the Huber regressor.

These facts attest to the homoscedastic error behaviour and negligible bias throughout the range of the yields. A synthesized analysis of the performance of the regression models is shown in Table 3.

**TABLE 3:** Comparative evaluation of contamination classification models showing accuracy and AUC-ROC, highlighting the superiority of the soft voting ensemble approach.

Model	R^2^	RMSE (FPU/mL)	MAE (FPU/mL)
Ridge regression	0.9699	0.0980	0.0694
Bayesian ridge	0.9997	0.0102	0.0072
Huber regression	0.9998	0.0085	0.0050
PLS regression	0.9937	0.0449	0.0310
Gradient boosting	0.9163	0.1634	0.1240
Extra trees	0.8864	0.1903	0.1492
XGBoost	0.6074	0.3539	0.2437
Stacking ensemble	0.9753	0.0888	0.0695
Blended ensemble	0.9703	0.0973	0.0739

The overall comparative evaluation results confirmed that although there are both complex ensemble models and robust models, linear models with strong regularization achieved higher results in the benchmark environment. This is an observation that indicates the proposed domain-informed feature engineering has effectively converted the original fermentation variables into a structured feature space, where meaningful biological relationships could be effectively captured without having to be highly flexible non-linear models. As a result, the Huber Regression and the Bayesian Ridge Regression methods were the ones with the best benchmark performance, and the PLS, Regression method is an important multivariate baseline to assess the latent-variable modeling of correlated fermentation features.

### 3.5 Performance of contamination risk models

The contamination risk prediction models were tested using accuracy, precision, recall, F1-score and AUC–ROC to measure the accuracy on the overall classification performance and the classification ability between the contaminated and non-contaminated fermentation runs. Given the moderate imbalanced class distribution (573 non-contaminated and 427 contaminated observations) a set of complementary evaluation metrics were taken into account instead of just the overall classification accuracy.

Gradient Boosting was found to be the highest standalone accuracy (83.0%), AUC-ROC (0.9009) which is high in discriminative ability. Logistic Regression and Random Forest classifiers were equally accurate but had lower recall or AUC scores, which implies that they are less sensitive to contaminated runs. The soft voting ensemble classifier showed the most promising total values, the accuracy was 83.5% and AUC-ROC value was highest 0.9045, as seen in Table 4. The ensemble method lowered variance and enhanced resistance to noise and imbalance of classes by summing probabilistic results of several different classifiers. The ensemble model achieved the best overall balance between discrimination and classification performance, providing the highest accuracy (83.5%) and AUC–ROC (0.9045). These results suggest that combining multiple classifiers improves robustness within the benchmark dataset by reducing model variance and enhancing sensitivity to contamination events. Although the improvements over the individual tree-based models are modest, they demonstrate the benefit of ensemble learning for contamination risk prediction in structured fermentation benchmark data. These findings support the notion that model diversity and the explicit treatment of imbalance is important in contamination detection, and the significance of ensemble strategies in predicting process-failure in bioprocess systems. The ROC curve is represented in Figure 9.

**TABLE 4:** Classification performance metrics after SMOTE balancing, summarizing accuracy, precision, recall, F1-score, and AUC-ROC for contamination detection models.

Model	Accuracy	Precision	Recall	F1-score	AUC–ROC
Logistic regression	0.8100	0.7195	0.7973	0.7564	0.9013
Random forest	0.8100	0.7432	0.7432	0.7432	0.8762
XGBoost	0.8150	0.7467	0.7568	0.7517	0.8778
Gradient boosting	0.8300	0.7632	0.7838	0.7733	0.9009
**Voting ensemble**	**0.8350**	**0.7733**	**0.7838**	**0.7785**	**0.9045**

**FIGURE 9:**
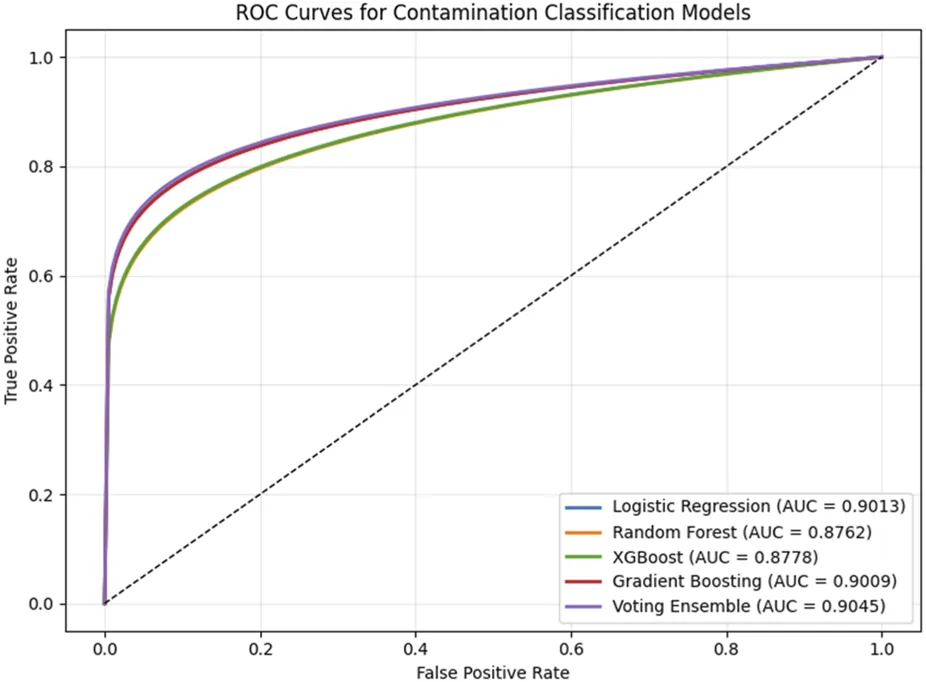
ROC curves for contamination-risk classification models, demonstrating discriminative capability across Logistic Regression, Random Forest, XGBoost, Gradient Boosting, and Voting Ensemble classifiers.

A naive majority-class baseline was also taken into account for contamination-risk prediction. The majority predictor has the potential to perform well in terms of nominal accuracy, but completely fail to detect contamination events that are rare in the dataset. Considering the limitation, recall, F1-score and AUC-ROC were found to be more informative measures for risk-aware monitoring of fermentation.

Given the nature of contamination detection (asymmetric misclassification cost), overall accuracy was not the only criterion used to evaluate. The main focus was on the recall, F1 score and AUC–ROC measure for securing appropriate sensitivity towards contamination events. Moreover, all the reported metrics were based on the original held out benchmark test set; and SMOTE was only used for the training partition. This evaluation protocol has avoided any information leakage and is free from any bias towards the performance of classifiers.

From an operational point of view, false negative contamination predictions can be much more expensive than false positives since a batch that is not contaminated but still fails may result in loss of resources, process instability downstream, or loss of batch. Thus, it is especially crucial to use recall-oriented evaluation in fermentation risk monitoring systems. Future work may further investigate cost-sensitive learning strategies and economic impact analysis using experimentally acquired fermentation datasets.

The proposed structure allows the study of trade-offs between process safety and productivity improvement by assessing yield forecast and process contamination risks in a combination and similar case. Joint evaluation of cellulase yield prediction and contamination risk enables simultaneous assessment of productivity and operational reliability within the benchmark environment. Rather than optimizing enzyme yield alone, the proposed framework provides complementary predictions that allow potential trade-offs between productivity and contamination risk to be explored during scenario analysis. This demonstrates the value of integrating regression and classification models within a unified surrogate modeling framework.

Overall, the contamination classification results indicate that ensemble learning provides the most consistent benchmark performance among the evaluated classifiers. When combined with the regression models presented in Section 3.4, the framework supports comprehensive benchmark evaluation of fermentation operating conditions by jointly considering productivity and contamination risk. Validation using experimentally acquired fermentation datasets will be required to establish the applicability of these findings to real bioprocess systems.

### 3.6 Surrogate framework and dashboard implementation results

In order to transform the trained models into a workable decision-support system the predictive pipeline was released as an interactive Cellulase Fermentation Surrogate framework dashboard. The dashboard is a user interface built around ingestion of data, training of models, performance metrics, simulation of scenarios and querying with AI support all working in the same integrated space. Figure 10 gives a summary of the surrogate framework system and operations status on the Home tab. It reports on the success of data loading, training models, and the system is available to make predictions. This page serves as a point of entry of users and conveys system availability in a very intuitive way.

**FIGURE 10:**
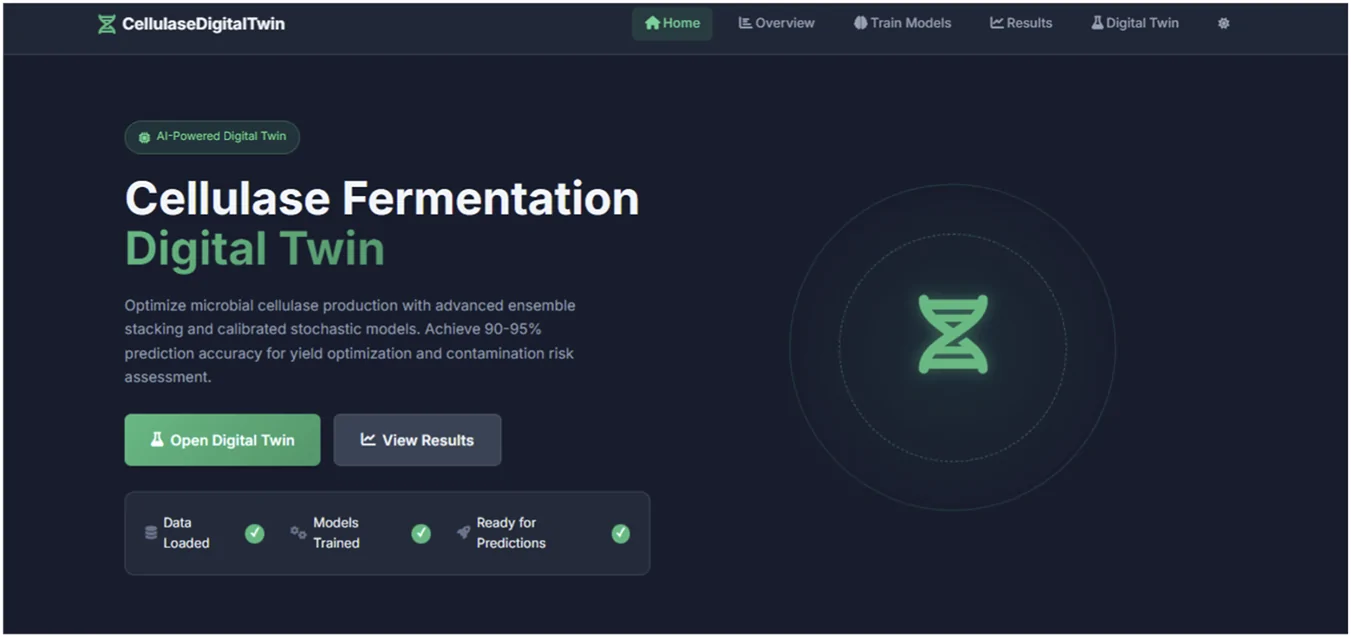
General tab of Cellulase Fermentation Surrogate framework dashboard showing system status, model preparation, and overview of the operation.

An overview tab, provides the statistical summary of the uploaded fermentation data. The important data features, such as the total sample number, engineered features, average yield, and the rate of contamination are presented with the yield statistics, including minimum, average, and maximum cellulase activity. The tab also displays average process parameters (temperature, pH, and dissolved oxygen) and offers a tabular preview of individual fermentation runs, which allows one to quickly examine the quality and distribution of data.

The Train Models tab allows the machine learning models described in the previous sections to be trained on-demand. The user is able to start training with the engineered feature set and the size of the dataset, the number of features, the approximate training time, and the type of regression and classification models that make up the pipeline are displayed as real-time indicators. This tab allows filling the gap between offline model development and interactive experimentation where users can retrain models when new data are available. Viewed together the Results tab summarizes the outputs of model evaluation, such as model regression and model classification metrics, model residual diagnostics, models feature-important visualization. These findings indicate that there is consistency in standalone evaluation and dashboard deployment. The main interactive element of the system is the Surrogate framework Simulator tab as represented in Figure 11. Slider-based fermentation controls allow the user to set the parameters of fermentation by temperature, pH, dissolved oxygen, agitation speed, inoculum size, lactose feed, duration, and media volume. When implemented, the system will produce real-time forecasts of cellulase production and the risk of contamination required by the functional conditions chosen. Predicted outputs are smooth and consistent to changes in the parameters, thus proving the generalisation and stability of the trained models.

**FIGURE 11:**
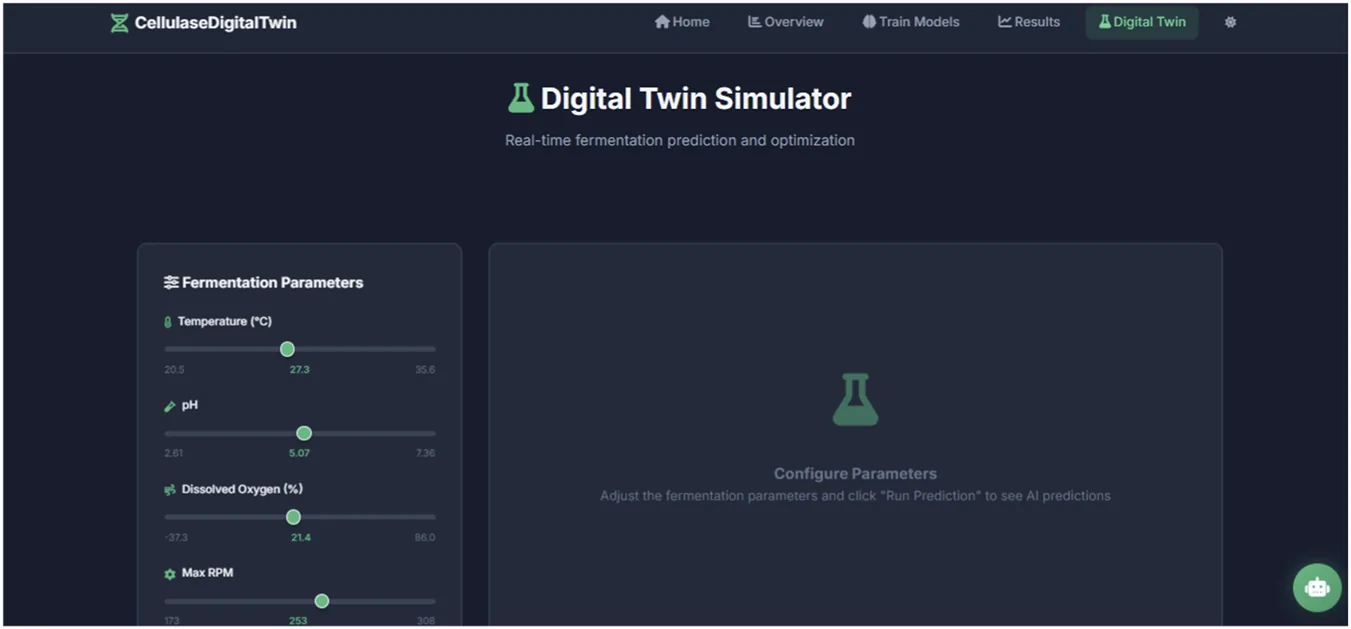
Real-time prediction output from the Surrogate framework Simulator displaying predicted yield and contamination risk based on selected parameters.

Moreover, the dashboard allows uploading datasets, which gives the capacity to analyze new fermentation data without having to modify the underlying codebase. This quality helps to make the system more extensible and facilitate the continuous learning processes. The usability of the surrogate framework can also be improved by an integrated Fermentation AI Assistant, that allows communicating with the system in a natural language. The assistant allows users to ask questions regarding the best ways to operate, ways to improve yields, prevent contamination, model choice, and sensitivity to parameters. Using trained model outputs and planned feature insights, the assistant uses what is known as context-aware responses which serve as a clever bridge between the domain specialists and predictive models.

The addition of the AI assistant makes the surrogate framework not just a passive visualization tool, but an active decision-support system, which provides an exploratory analysis and helps to test the hypothesis and optimize fermentation faster. Simulations on the basis of dashboards demonstrated high correlation with offline assessment outcomes, as yields and contamination likelihood were nearly the same as in case of a static test. This stability proves that the trained models do not lose their predictive fidelity to deployment in an interactive and real-time environment.

Altogether, the Cellulase Fermentation Surrogate framework shows that highly developed machine learning models can be implemented into a convenient, interactive interface where fermentation optimization can take place. Through the combination of predictive model, risk evaluation, and AI-based collaboration, the suggested system offers a universally applicable and explainable framework of data-driven bioprocess decision-making.

## 4 Discussion

This work highlights the benefits of domain knowledge driven feature engineering and the use of suitable machine learning models for attaining a high level of accuracy in a systematic fermentation benchmark. Huber Regression had the best benchmark performance (R^2^ = 0.9998) followed closely by Bayesian Ridge Regression (R^2^ = 0.9997) and Partial Least Squares (PLS) Regression also had a good predictive performance (R^2^ = 0.9937). The observation that after biological-to-surrogate descriptor transformation, the engineered feature space has a structure that is well suited to robust linear and latent-variable regression models, indicates that these models have the potential to predict accurately the protein’s biological activity. Tree-based ensemble methods on the other hand, did not yield as high predictive performance, indicating that many of the nonlinear relationships were already learnt through the extensive feature engineering already done. Not all models will necessarily perform better on the benchmark, however, as the model’s complexity increases.

The regression ability is very high, but this is not an indication of the ability of the industry to predict. The benchmark has a hybrid dataset of 20 fermentation observations acquired in the lab, along with 980 samples synthetically generated, and the FERM-AI preprocessing pipeline also reconstructs both the prediction targets using domain knowledge-based mathematical formulations before training the model. The characteristics of these controlled benchmarks minimize the random variation of measured characteristics and enhance the controlled relationships between engineered features and prediction targets. Thus, reported R^2^ values indicate the capacity of the proposed surrogate modeling framework to retrieve structured benchmark relationships in the context of repeatable experiments and not in the context of independent industrial ferments data sets. This distinction is crucial to the proper evaluation of the reported predictive accuracy.

This is important because the use of PLS Regression is common in chemometrics and bioprocess engineering for the modelling of correlated process variables, and for limited sample size, yields an important benchmark for comparison. Despite the slightly less predictive accuracy of PLS, it was found to be very effective and it is a testament to the strength of latent-variable approaches that it can work well with datasets that have a high degree of multicollinearity, such as those found in fermentation. The comparative evaluation thus shows that the available set of powerful linear, probabilistic and latent-variable regression techniques offer good baseline for the prediction of fermentations.

The proposed framework not only models the yield of cellulase, it also models the contamination risk, where it is a complementary binary classification task. The training partition is moderately imbalanced (573 non-contaminated and 427 contaminated observations), therefore, SMOTE was only applied to the training partition for the benchmark dataset. Multiple probabilistic classifiers gave a higher classification performance in terms of the accuracy (83.5%) and AUC–ROC (0.9045), suggesting that the ensemble gives a higher robustness and discrimination than the individual classifiers. Furthermore, measuring overall accuracy along with recall, F1-score, and AUC–ROC offers a more informative way of measuring contamination detection than just accuracy, since contamination events are the ones that would impact the most practically.

An important aspect of the proposed framework is the incorporation of prediction of productivity and contamination risk in a single surrogate modeling framework. Most of the previous machine learning works on the fermentation process have only considered the maximization of enzyme productivity, but the present work considers both enzyme productivity and operational reliability. The benchmark data are not suitable for quantitative optimization of the industrial operating strategies, but the application of the combined regression–classification framework shows the potential for scenario-driven analyses to investigate the different scenarios that can be utilized to explore potential trade-offs between enzyme yield and the risk of contamination. It is a dual objective formulation that offers a more complete set of guidelines for assessing machine learning methodologies, beyond just prediction of productivity.

The engineered feature representation also significantly affects the model performance and interpretability. The proposed framework is not based solely on the raw variables of the fermentation process, but also on mathematically defined variables to describe the aspect of optimality of the fermentation process, the aspect of environmental stress, the aspect of substrate utilization, the aspect of nonlinear interactions, and the aspect of normalized operating conditions. The weakened predictive signals in the benchmark data were reinforced by the biologically motivated transformations, as these consistently appeared as one of the largest feature importance predictors in the feature importance analysis. The above-mentioned engineered descriptors must, however, be viewed as surrogate descriptors to facilitate the learning of the benchmark, and not as experimentally proven mechanistic descriptors.

The present study intentionally leaves out mechanistic and hybrid bioprocess modeling but includes only the data driven surrogate modeling. The benchmark dataset is lacking the detailed temporal information about the processes, biomass dynamics, substrate consumption profiles, and information about the control of the reactor that is required for classical kinetic models based on Monod-type equations or hybrid first-principles/machine learning models. Since the available data are rather limited in the amount of available fermentation trajectory information as well as the number of complete data sets, the development of mechanistic Digital Twin models was not covered in this work. The use of mechanistic models with machine learning in the future could provide biological interpretability and provide the ability of process simulation for processes where experimental datasets are available.

It is necessary to take into account several restrictions in interpreting the results reported. First, the benchmark has 20 fermentation runs that are acquired in the experiments, but most of the observations in the benchmark are synthetically generated and the target variables are reconstructed in the preprocessing. Thus, there was no possibility for independent validation with only the fermentation data acquired from experiments. The proposed framework should therefore be considered a framework based on a benchmark, which represents a surrogate modelling approach rather than an actual predictive system for industry or a closed-loop Digital Twin. Further validation on larger sets of experimental data, pilot-scale experiments in bioreactors and industrial fermentation runs will be needed to consider practical deployment.

Second, the benchmark data set only includes endpoint values of the process descriptors and does not provide any temporal information on fermentation trajectories, biomass growth, consumption of substrates, oxygen uptake rate, carbon evolution rate or other temporal measurements that are required for dynamic process modeling. Thus, the proposed model does not model the total temporal evolution of the fermentation process, but rather only the endpoint cellulase productivity and the risk of contamination. The real-time sensor data and real-time dynamic bioprocess measurements are important future research opportunities. Last but not least, there are a number of methodological drawbacks. While the engineered feature representation showed good predictive power, a formal ablation study which quantified the power of each feature category was not performed here. Similarly, between the competing machine learning models, a statistical significance test was not performed on them, and repeated cross validation and precision–recall curve evaluation were not done. These analyses would also give more evidence of the robustness of the models and comparative performance and would be valuable benchmarks for future investigations.

Overall, the results show that the FERM-AI framework is a transparent and reproducible surrogate modeling framework for comparing machine learning methods for prediction of cellulase fermentation. The framework allows for biologically motivated feature engineering, powerful regression methods, latent-variable modelling, contamination-risk classification and interactive scenario analysis, enabling a comprehensive assessment of machine learning methods in controlled settings while making it clear what is considered a machine learning “benchmark” and what is an application for industry.

## 5 Conclusion

A benchmark-oriented surrogate modeling framework for cellulase fermentation was presented in this study, called FERM-AI, and the framework is designed to incorporate the prediction of enzyme yield, the assessment of the risk of contamination, and the interactive analysis of scenarios in a single machine learning process. The proposed framework is built with domain-informed feature engineering, powerful regression model, latent-variable modelling, ensemble classification and explainable machine learning, which offers a reproducible framework for benchmarking the various predictive modelling strategies using a publicly available benchmark fermentation dataset.

Huber Regression had the best benchmark performance (R^2^ = 0.9998) followed closely by Bayesian Ridge Regression and the good latent-variable baseline was offered by Partial Least Squares (PLS) Regression. The ensemble classification performance was the best when only SMOTE was used on the training data, showing that using both productivity prediction and contamination-risk assessment is a more complete measure of classification performance than productivity prediction alone when used for contamination risk prediction. These results need to be considered in the frame of the structured benchmark dataset and show the efficacy of the proposed surrogate modeling methodology, but cannot be interpreted as a validated predictive performance of the proposed methodology for an industrial fermentation system.

The proposed framework also highlights the potential of biologically inspired feature engineering and interactive visualization to help in transparent investigation of the operating parameters of fermentation processes and comparative analysis of machine learning models under well-controlled benchmark conditions. FERM-AI is not a validated industrial Digital Twin but is a valuable platform for comparison of surrogate modeling techniques and study of the relationship between cellulase productivity and contamination risk that can be easily extended and interpreted by the research community.

In future, the framework will be validated with larger sets of fermentation data to be acquired from experiments, time-series bioprocess measurements will be integrated with real-time sensor data, hybrid models of mechanistic–machine learning will be explored, and statistical significance analysis of attributes, feature ablation and multi-objective optimization approaches will be explored. Together, these will enhance the biological realism and practicality of AI supported fermentation modelling and ensure the transparency and reproducibility that has already been developed in the present benchmark framework.

## Data Availability

The raw data supporting the conclusions of this article will be made available by the authors, without undue reservation.
